# Experiences of Care in Intensive Care Units After a Suicide Attempt From the Perspectives of Patients, Family Members and Healthcare Personnel—A Scoping Review

**DOI:** 10.1111/nicc.70556

**Published:** 2026-06-26

**Authors:** Aleksandra Jarling, Fredrika Sundberg

**Affiliations:** ^1^ Faculty of Caring Science, Work Life and Social Welfare University of Borås Borås Sweden; ^2^ PreHospen Centre for Prehospital Research University of Borås Borås Sweden; ^3^ Research, Education, Development and Innovation Department Skaraborg Hospital Skövde Sweden; ^4^ The School of Health Sciences University of Skövde Skövde Sweden

**Keywords:** caring, critical care, experiences of care, intensive care unit (ICU), patients and family members, scoping review, suicide attempt

## Abstract

**Background:**

Suicide attempts can lead to life‐threatening conditions that require intensive care. In such situations, patients often experience existential fear when cared for in highly technological and unfamiliar environments such as the intensive care unit. The circumstances surrounding the suicide attempt, together with their critical condition, may further influence their ability to cope and shape their experience of care. Understanding these experiences is important to ensure that the therapeutic relationship supports the recovery process. Interprofessional collaboration and communication are essential for coordinated, recovery‐oriented care.

**Aim:**

To map the existing evidence on how patients, family members and healthcare personnel experience care in intensive care units following a suicide attempt, to identify key themes, knowledge gaps and implications for clinical practice and future research.

**Study Design:**

This scoping review was conducted using the Arksey and O'Malley methodology and the PRISMA‐ScR list. Six databases were systematically searched using predefined keywords. All English‐language studies addressing the review aim were included.

**Results:**

The search yielded 1107 articles. After screening, 13 were assessed in full, and five met the inclusion criteria. These studies, published between 1985 and 2011, comprised four surveys and one qualitative interview study. Findings indicate that patients felt misunderstood, families lacked information and support, and personnel experienced emotional strain. Attitudes varied widely, and healthcare personnel expressed a need for further education and reflective practice in suicide care.

**Conclusions:**

Only five relevant studies were identified, all published between 1985 and 2011, with just one including patient or family perspectives. This limited and dated research highlights a clear gap in research on intensive care following suicide attempts and precludes firm conclusions about care experience. With these constraints, the findings tentatively suggest the importance of compassionate, holistic and collaborative approaches to care, as well as the potential value of personnel's support and training and policies that attend to the mental health needs of suicidal patients and their families.

**Relevance to Clinical Practice:**

The findings may suggest the potential relevance of a holistic approach to caring for patients after a suicide in ICU practice, where physical, psychological, social and emotional aspects of care are considered in supporting recovery and well‐being.

## Impact Statements

- What is known about the topic
  ○ Suicide is a prevalent global health issue, and many individuals who attempt suicide require advanced, life‐saving treatment in the ICU.
  ○ Patients admitted after a suicide attempt often experience severe psychological distress and therefore require empathetic and supportive interactions from healthcare professionals.
  ○ The highly technological and intensive environment of the ICU can pose additional challenges for these patients, influencing their emotional state and overall experience of care.
- What this paper adds
  ○ This study underscores the marked scarcity of research and limited development of expertise in this area, highlighting an urgent need for further investigation and targeted educational initiatives.
  ○ This study provides an initial glimpse into the vulnerability experienced by patients, family members and healthcare personnel in the context of ICU care following a suicide attempt.
  ○ This study suggests that gaps in knowledge and uncertainty among healthcare personnel may be associated with countertransference reactions, potentially influencing how care is experienced and delivered.

## 1 Introduction

Mental health problems are a global crisis, with many individuals struggling to find meaning in life. Suicide, defined by the WHO [1] as the intentional act of ending one's life, is often the result of a combination of factors, including vulnerability and suffering. Suicide rates are increasing, with nearly 1 million deaths by suicide annually, and Europe having the highest rates, with several EU countries exceeding 15–20 deaths per 100 000 population [2]. In the United States, the 2022 suicide rate was 14.2 per 100 000 [3, 4]. For every suicide, many more attempt it, with previous attempts being a key risk factor. Suicidal behaviour is a serious mental health issue, and its high comorbidity with other psychiatric conditions complicates the assessment and management of risk factors [5]. The highest risk is among individuals in low‐ and middle‐income countries, particularly men, though women are more likely to attempt suicide [1].

Suicide attempts can result in life‐threatening conditions requiring intensive care. Intentional self‐harm is a common reason for being admitted to intensive care unit (ICU) [6], with medication overdose being the most frequent method of suicide attempts [7]. Approximately 3.6%–5.5% of all ICU admissions are due to self‐harm or suicide attempts, with the proportion rising in recent years. Among patients visiting the emergency department for suicide attempts, around 16.8% are admitted to an ICU, with many requiring treatments for severe medication overdose or violent injury [6, 8]. The average length of stay in ICU after a suicide attempt varied, with a mean duration of 2.43 days (1–11 days); approximately 60% are women [9, 10]. After being discharged from the hospital following a suicide attempt, the risk of a subsequent attempt increases significantly, by up to 300 times [11]. This risk is particularly elevated among ICU survivors aged 18 to 34 and those with pre‐existing mental illnesses [12].

A serious suicide attempt affects a person's entire existence, often hovering between life and death, and is followed by feelings of emotional pain and disconnection [13], characterised by high levels of hopelessness, sorrow and suffering [14]. Such distress may hinder patients' ability to express themselves, and communication of emotional pain is influenced by multiple factors. Some patients may refrain from speaking due to fears of not being taken seriously by healthcare personnel [15].

In parallel, critically ill patients in ICUs receive advanced medical and nursing interventions involving continuous monitoring, complex treatments and sophisticated medical technology [16]. The ICU environment is therefore highly technological and shaped by the presence of such equipment [17, 18, 19, 20, 21]. While these technologies enhance patient safety, they can also demand substantial attention from healthcare personnel, potentially limiting time for direct patient interaction [20]. Moreover, the ICU environment may expose patients to constant stimuli that increase stress, disrupt sleep and circadian rhythms, and contribute to complications such as ICU delirium [21, 22, 23].

Within this context, patients may experience heightened existential fear as they face life‐threatening conditions while being cared for in an unfamiliar, technology‐intensive environment [24, 25]. Addressing this vulnerability requires attentive, compassionate care and support from both healthcare professionals and family members. Families can play an important role in recovery and should be recognised as valuable contributors to care processes [26]. Altogether, receiving ICU care following a suicide attempt is a complex experience that warrants consideration from multiple perspectives. Therefore, this scoping review aimed to map existing evidence on how patients, family members and healthcare personnel experience ICU care after a suicide attempt to identify key themes, knowledge gaps and implications for clinical practice and future research.

## 2 Methods

### 2.1 Study Design

A scoping review was conducted following the framework originally proposed by Arksey and O'Malley [27] and further refined by the Joanna Briggs Institute (JBI) [28]. While Arksey and O'Malley provide the foundational methodological structure for scoping reviews, JBI offers more detailed and updated guidance to enhance methodological rigour and transparency. Combining these approaches is consistent with current best practice in scoping review methodology. The review followed the PRISMA‐ScR (Preferred Reporting Items for Systematic Reviews and Meta‐Analyses extension for Scoping Reviews) guidelines and checklist [29].

### 2.2 Inclusion and Exclusion Criteria

Eligibility criteria were guided by the Population–Concept–Context (PCC) framework [30], as recommended by JBI. The *Population* comprised patients, family members and/or personnel; the *Concept* encompassed experiences following a suicide attempt; and the *Context* focused on care within an ICU. All experiences related to suicide attempts were considered regardless of method, including but not limited to intoxication, self‐harm, hanging or other forms of bodily injury with expressed or suspected intent to end one's life.

This review included original, peer‐reviewed studies published in English, employing qualitative, quantitative or mixed methods study designs. The literature searches were conducted without date restrictions, covering all records available in each database from inception up to the search date. Studies that described patients' experiences of being cared for in the ICU after a suicide attempt, as well as family members' and healthcare personnel's' experiences of the ICU were included. Articles were included regarding all ages of the patients and only those being cared for in an ICU. This could include both ICU and paediatric ICU (PICU). Grey literature was excluded to ensure the inclusion of peer‐reviewed studies with established scientific quality, and articles addressing assisted suicide or experiences of care in psychiatric intensive care units were also excluded.

### 2.3 Search Strategy

The search strategy was developed in collaboration with two experienced health sciences librarians, one from a university and one from a hospital. They were consulted and guided the search process and the development of inclusion and exclusion criteria, incorporating both controlled vocabulary (e.g., MeSH terms) and free‐text keywords.

### 2.4 The Search

A systematic database search was conducted in a structured manner on 26 May 2025. Six different databases were included in the search: CINAHL, PubMed, Web of Science, PsycINFO, the Psychology Database and PsycARTICLES. Search terms were used in different variations of the search (Table 1).

**TABLE 1:** Overview of the search strategy used in each database.

Search terms				
suicid* [title/abstract]	AND	intensive care unit OR icu OR critical care OR critical care unit [title/abstract]	AND	experiences OR perceptions OR attitudes OR views OR feelings OR qualitative OR perspective [title/abstract]

**FIGURE 1:**
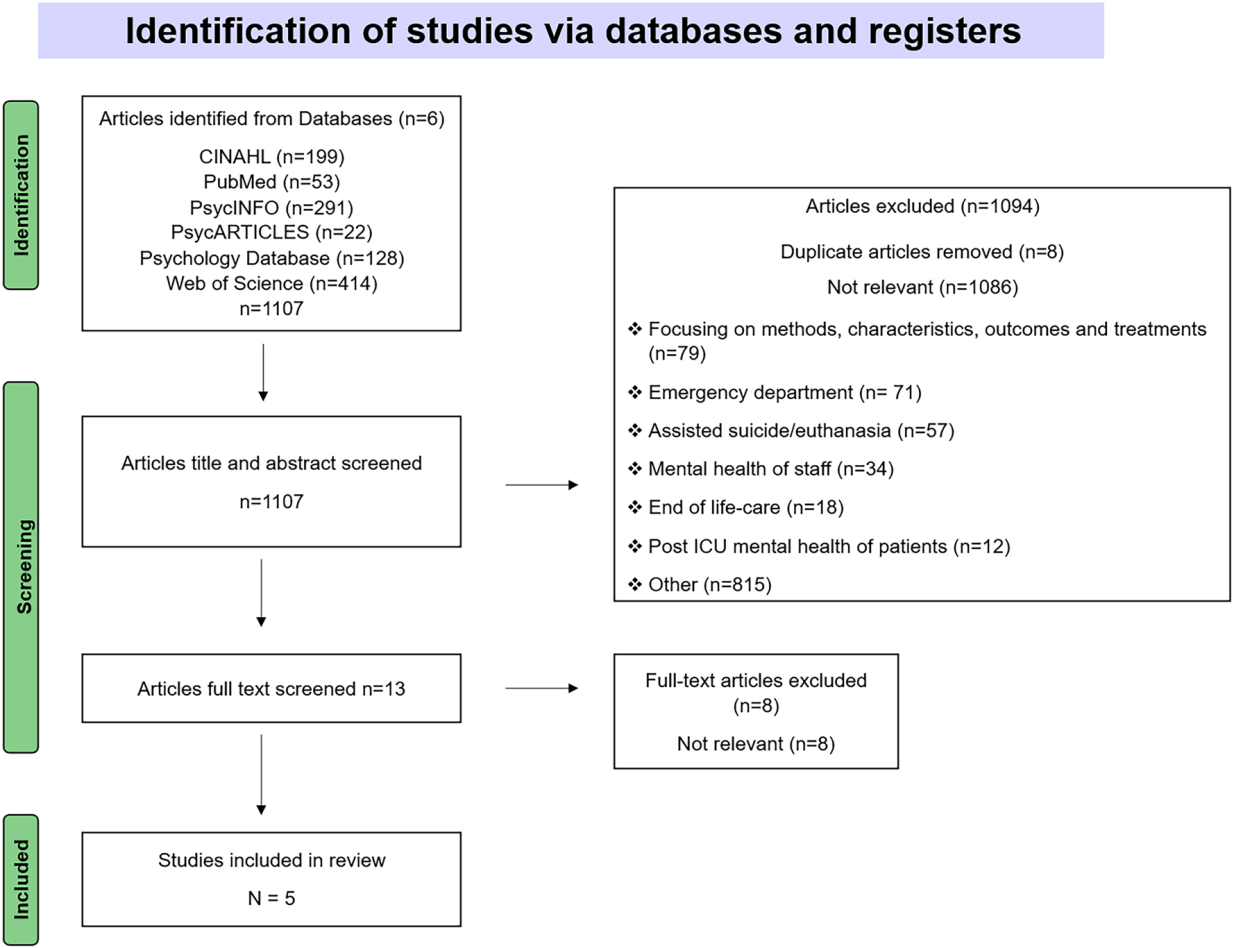
Flow diagram of study selection.

This process was conducted initially by reviewing titles, followed by abstracts and finally full‐text screening. The selection process involved the two independent researchers who screened titles, abstracts and full texts. In addition to disagreements, questions, uncertainties and doubts also arose during the process, leading to an in‐depth discussion between the researchers and sometimes with colleagues who possess specialised expertise in the field. This collegial dialogue helped to refine perspectives and deepen the understanding of the complex issues addressed in the work. The search strategy is reported in the PRISMA flow chart diagram (Figure 1).

### 2.5 Critical Appraisal

A quality appraisal was conducted to provide contextual insight into the robustness of the included studies. The JBI critical appraisal tools, tailored to the specific study designs, were used for this purpose and presented in Table 2.

**TABLE 2:** Included articles.

No.	Article	Country	Aim	Method	Critical appraisal
1	Bailey (1994) [31]	Australia	To investigate the attitudes of critical care nurses and doctors to parasuicide patients.	A descriptive survey study using a convenience sample with 299 nurses and 81 doctors (*N* = 380). Data obtained from responses to a Likert‐type questionnaire were assessed.	7/8^a^
2	Kishi et al. (2011) [32]	Japan	To examine the attitudes among Japanese nurses together with their perceived need for training in relation to understanding the nature of suicidal behaviour and preventive strategies.	The Understanding Suicidal Patients (USP) scale together with additional questions reflecting training and the psychiatric treatment of suicide attempters were administered whereof 323 nurses attended this study. The fields of nursing practice were divided into the following four categories: general medical/surgical unit, emergency care/intensive care unit, psychiatric unit and other.	6/8^a^
3	Lönnqvist and Suokas‐Muje (1986) [33]	Finland	To determine the attitudes of the staff in an out‐patient department and intensive care unit in a general hospital, and the attitudes of the staff in a psychiatric hospital, towards patients who attempt suicide.	A descriptive survey study using sample with 251 staff members. Whereas 166 nurses and doctors from the out‐patient department and an intensive care unit and 85 staff members from the Department of Psychiatry (psychiatric hospital). Data obtained from responses to a Likert‐type questionnaire were assessed.	6/8^a^
4	Suokas and Lönnqvist (1989) [34]	Finland	To explain the attitudes of emergency personnel (*N* = 184) towards patients who attempt suicide in the different stages of treatment in a general hospital by comparing the attitudes of the staff in the emergency room (*n* = 64), emergency ward (*n* = 47) and intensive care unit (*n* = 73).	A descriptive survey study using a sample with 184 nurses and doctors from three units. Data obtained from responses to a Likert‐type questionnaire were assessed.	6/8^a^
5	Wolk‐Wasserman (1985) [35]	Sweden	To describe the suicide attempt patients' and their relatives' experiences and reactions during the patients' stay on the intensive care unit. In addition, to survey the feelings and reactions of the personnel which arose during confrontations with the suicide attempt patients.	A qualitative interview study was performed. Semi‐structured interviews with 40 patients were carried out, first in connections with their admission and once again during the following year, a total of 88 interviews. The patients' significant others (*n* = 70) were interviewed during the first week and once again during the following year, a total of 129 interviews. Semi‐structured with 22 personnel (physicians, nurses and nurse aides), were performed.	10/10^b^

^a^ JBI Critical appraisal checklist for analytical cross sectional studies [36].

^b^ JBI Critical appraisal checklist for qualitative research [37].

### 2.6 Data Extraction

Data were extracted using a structured charting form based on JBI templates and analysed using descriptive methods, complemented by a cautious, inductive thematic synthesis that remained closely grounded in the limited data. In accordance with Stage 5 of the Arksey and O'Malley scoping review framework, data were collated and summarised into three categories [27]. Guided by the review aim, the Results sections of the included studies were examined to extract data on experiences of intensive care following a suicide attempt, which were compared across studies. Recurring patterns were grouped into overarching categories to provide a thematic mapping of the evidence rather than a formal synthesis.

## 3 Findings

The search process identified a total of 1107 articles, including duplicates (Figure 1). All titles and abstracts were reviewed for relevance in accordance with the study's objectives and the PRISMA guidelines [28]. Records not being coherent with the aim of the study were excluded. A substantial number of the excluded studies primarily focused on the specific methods by which patients attempted suicide and their respective treatment regimens, rather than focusing on the provided care within the ICU. The category ‘other’ (Figure 1) included conference papers, editorials and studies with a community interest, such as mental health of veterans, members of the LGBTQI+ community, maternal/postpartum health and patients living with HIV. Following this initial screening, 13 articles were selected for full‐text review. Of these, eight articles were excluded prior to quality appraisal because their scope was not coherent with the aim of this study. Consequently, five articles were included in the review.

The five included studies were published between 1985 and 2011 (Table 2), with three originating from the 1980s [33, 34, 35]. Notably, these three studies shared a common Scandinavian context, while the others originated from Australia [31] and Japan [32]. All five studies focused on the nurses' and other healthcare personnel's perspectives (*n* = 1160), with only one incorporating the experiences of patients (*n* = 40) and their family members (*n* = 70) [35]. All the included studies focused on adult populations. A descriptive study design employing surveys was the most common approach [31, 32, 33, 34], with only one study adopting a qualitative approach using interviews [35].

### 3.1 Patients' Experiences of Care in the ICU After a Suicide Attempt

While studying lived experiences in the ICU, Wolk‐Wasserman [35] described how patients upon regaining consciousness reported physical pain from life‐sustaining medical devices and were generally satisfied with somatic care. However, the patients did not anticipate attention to their mental health needs, perceiving personnel as extensions of medical technology rather than individuals. This perception reinforced the belief that personnel could not understand their emotional distress and existential crisis. Patients wanted and needed to discuss their emotions, which personnel often dismissed. Some patients reached out and sought help to talk about their feelings, while others did not and remained silent. They experienced discomfort and a sense of being monitored when personnel observed them without engaging in dialogue, leading to confusion about the lack of interaction, especially when perceived to be at high risk of suicide.

### 3.2 Family Members' Experiences of Care in the ICU

In this one study, family members of patients in intensive care described the experience as profoundly distressing, representing one of the most challenging periods in their lives. This group, including partners, parents, children, and other relatives and friends, expressed a strong need to discuss both their own circumstances and those of the patient during visits. However, they lacked access to private spaces for undisturbed conversations with the patient or personnel, who themselves had limited time for such interactions and emotional support. As a result, family members felt vulnerable and sometimes perceived the personnel's communication as accusatory. They felt isolated and expressed a need to discuss their situation. Adult children felt burdened by responsibility for the suffering parent and preferred the hospital to take over, being demanding towards personnel and critical when expectations were unmet [35].

### 3.3 Personnel's Experiences and Perspectives

ICU personnel caring for patients after suicide attempts reported emotions such as powerlessness, hopelessness, insecurity, fear, anxiety and guilt, often intensified by identifying with patients or relatives [35]. Personnel who identified with patients tended to show greater empathy and warmer contact, whereas others distanced themselves from patients they found difficult to understand. Conversations with suicidal patients could lead to personal involvement, which personnel struggled to handle, resulting in sometimes reserved interactions [35]. Some personnel perceived these patients as diverting resources from those considered in greater medical need [33, 34].

Attitudes towards patients varied: some expressed sympathy, others irritation [32, 33]. Irritation and aggression were common towards patients seen as manipulative or ungrateful, especially those with repeated suicide attempts [31, 35]. Over half of the respondents viewed parasuicide patients as manipulative, and most were unconcerned about saying the ‘wrong thing’ [31]. Many believed these patients should be treated in specialised wards with psychiatric expertise [35]. ICU nurses were less likely than general unit nurses to understand or sympathise with suicidal patients and less inclined to discuss emotions [32]. Although personnel wanted more opportunities to reflect on their reactions and gain better insight into patients' motives [35] and also requested further education on suicide care [31, 33].

## 4 Discussion

The systematic literature search for this study yielded only five relevant articles, of which only one included the experiences of patients and their family members. Notably, none of the included articles were published within the past 15 years, despite a comprehensive and rigorous search process across multiple databases using broad search terms. These limited results highlight a substantial and persistent gap in the research regarding experiences of care in the ICU following a suicide attempt. The scarcity of research likely reflects the profound ethical, psychological and methodological challenges inherent in studying this sensitive and complex population, but the absence of evidence remains consequential. Our dataset is small and uneven, with very few first‐person accounts from patients or families; therefore, claims are necessarily provisional and limited. Yet even within these constraints, the available literature provides some examples of potential shortcomings: mental health needs in ICU settings may not always be sufficiently addressed; patients in some cases describe being perceived primarily through technical tasks, with intensified monitoring not consistently accompanied by caring interaction or communication; and families in certain instances report unmet needs for communication and support [35]. Although the available research is very limited, it may suggest a few potential directions for further inquiry, including the role of psychological support within ICU contexts and aspects of communication with families. Overall, these findings should be interpreted cautiously and may help to inform future research on caring for individuals recovering from a suicide attempt in the complex ICU environment. The findings from this review suggest some areas for further exploration regarding ICU care for patients admitted after a suicide attempt, while acknowledging the constraints of intensive care practice. Given that many patients admitted to the ICU are sedated [38] or unconscious for significant parts of their admission, opportunities for direct therapeutic engagement are limited. Nevertheless, the reviewed studies highlight challenges related to communication, psychological support and personnel preparedness within intensive care settings. The findings suggest that greater attention to mental health in the care of individuals following a suicide attempt may contribute to more consistent care practices. Drawing on evidence‐based strategies described across diverse clinical settings [39]. These approaches may include attention to suicide‐risk indicators at admission, supportive communication when patients are awake and documentation of psychosocial concerns when identifiable. Such measures may help provide a clearer framework for personnel without necessarily requiring extensive psychological expertise [40]. For families, the reviewed studies indicate that clarity, continuity of information and clearly designated points of contact may at times be insufficient during ICU admissions. Structured and regular communication, together with clear written information, may support families' understanding of the care plan and help reduce uncertainty during ICU admissions [41].

A caring approach that considers the vulnerability of being human is essential, an approach grounded in and guided by an understanding of the patient as a whole, embodied being [42]. In intensive care, the body is entrusted to the care of others, and treatment is often based on standardised protocols. A profound understanding of suicide as an expression of unbearable suffering and an intolerable life situation, regardless of its underlying causes, is essential. Such understanding is, however, only meaningful if personnel themselves have developed this awareness and possess the necessary knowledge. In this context, personnel may serve as crucial resources, capable of reminding suicidal individuals of their inherent dignity and worth as human beings [43]. However, the process of daring to face oneself, accept the situation and find hope after a suicide attempt is difficult. It must begin early and be supported in all caregiving contexts [44]. Personnel in ICU, therefore, play a key role in the recovery and health process to suicide prevention.

The perspectives of personnel highlighted in the present study suggested complexities in caring for patients following suicide attempts. In some cases, patients were described in ways that reflected frustration among personnel, particularly in contexts characterised by high acuity, time pressure and limited opportunities for interaction. Such emotional responses may be interpreted in relation to the concept of *countertransference within psychoanalytic theory* [45]. Countertransference is often associated with patients who have emotionally unstable personality disorder (EUPD), where chronic suicide attempts are common [46]. Suicidal patients can provoke adverse countertransference in healthcare professionals, potentially influencing outcomes; therefore, personnel should recognise and manage these reactions to provide optimal care [47]. Despite these challenges, empathy and sympathy appeared to be more common among permanent personnel who identified with patients or their relatives, suggesting that greater understanding of the background and circumstances surrounding suicide attempts may be valuable in care encounters. Intensive care nurses have described limited preparedness in holistic care for patients with mental illness in the ICU. Additional training may contribute to greater awareness of stigma and negative attitudes towards vulnerable patients [48]. The challenges inherent in caring for patients with mental health disorders and complex clinical situations also highlight the role of collaboration between the ICU and psychiatric care in supporting continuity and patient‐centred care. Such collaboration may support the exchange of competencies, help address professional and organisational challenges, and contribute to a more integrated approach to care, in line with findings by Wilson et al. [49] who emphasise that support from the multidisciplinary team and mental health services is key in ensuring high‐quality care for ICU patients with mental health disorders.

During the writing process and literature searches focusing on caring in the context of ICU in relation to suicidal behaviour and care needs, it appeared that this issue was discussed more extensively in earlier literature [34, 48]. More recent studies on the subject are relatively limited. The findings may suggest that contemporary intensive care often places substantial emphasis on medical perspectives in the context of suicide attempts while psychosocial and individual care needs may receive comparatively less attention. This is reflected in research, describing how healthcare can at times be experienced as focusing on mechanised care and sustaining the physical body, rather than addressing the holistic needs of the individual.

### 4.1 Limitations

This review has several limitations that need to be acknowledged. Most notably, there were extremely few and dated studies that focused on the experiences of care in the ICU from patients and their families, following a suicide attempt, which limits the strength of the findings. Four of the five included studies that focused on the experiences of personnel utilised surveys as a method, which has the possibility of respondent bias that needs to be considered.

The search strategy may have been overly restrictive, as it ultimately yielded only five eligible articles. Although the initial search identified 1107 records, all titles and abstracts were screened independently by both authors. Thirteen articles were selected for full text review; however, only five addressed the experience or provision of care in the ICU following a suicide attempt. Additionally, the review deliberately excluded grey literature and was limited to English‐language, peer‐reviewed publications, which may have resulted in the omission of other relevant studies. Given the small number of included studies, the breadth and representativeness of the available evidence are limited.

Existing evidence from emergency departments highlights limited empathy and negative attitudes towards patients who self‐harm, and challenges in interacting with and responding to the patient. These findings may also have relevance for intensive care contexts, particularly in relation to education and support for personnel [50]. Overall, this limited body of evidence suggests that the experiences of care in an ICU after a suicide attempt from the perspective of patients, family members and healthcare personnel remains insufficiently explored. Notably, a study published earlier this year from Norway indicates growing scholarly attention to this area [51], suggesting that the topic may receive greater focus in future research.

## 5 Implications for Practice and Further Research

The findings point to shortcomings in addressing the mental health needs of both patients and their families. Patients often report feeling misunderstood, while the needs of family members may remain insufficiently recognized and addressed. Healthcare professionals, particularly nurses, may also experience countertransference, which can influence the quality of care provided. Together these findings suggest the importance of empathetic, and holistic approaches to care in intensive care settings and indicate that further research is needed to deepen understanding and improve care for this vulnerable patient group.

## 6 Conclusion

The systematic literature search identified only five relevant articles, none of which were published within the past 15 years, highlighting a significant gap in research on intensive care following suicide attempts. This limited body of research underscores the challenges of studying such a sensitive and complex topic. Given the limited number of available studies, the findings should be interpreted with caution and cannot be readily generalised. Nevertheless, the included studies may provide some insight into experiences of caring for, or being cared for in, an ICU following a suicide attempt. They also indicate that further research from a caring perspective could contribute to a broader understanding of this area, particularly considering the ongoing global burden of suicide and psychological suffering.

## Funding

The authors have nothing to report.

## Ethics Statement

The authors have nothing to report.

## Conflicts of Interest

The authors declare no conflicts of interest.

## Data Availability

The data that support the findings of this study are available on request from the corresponding author. The data are not publicly available due to privacy or ethical restrictions.

## References

[1] WHO , Suicide Worldwide in 2019 (World Health Organization, 2021).

[2] WHO , “Suicide Rates,” 2022, https://www.who.int/data/gho/data/themes/mental‐health/suicide‐rates.

[3] Centers for Disease Control and Prevention , “Suicide Data and Statistics | Suicide Prevention | CDC,” 2024, https://www.cdc.gov/suicide/facts/data.html.

[4] A. L. Cammack , M. R. Stevens , R. B. Naumann , et al., “Vital Signs: Suicide Rates and Selected County‐Level Factors—United States, 2022,” MMWR. Morbidity and Mortality Weekly Report 73 (2024): 810–818, 10.15585/mmwr.mm7337e1. 39298366 PMC11412441

[5] R. T. Liu , “The Epidemiology of Non‐Suicidal Self‐Injury: Lifetime Prevalence, Sociodemographic and Clinical Correlates, and Treatment Use in a Nationally Representative Sample of Adults in England,” Psychological Medicine 53, no. 1 (2023): 274–282, 10.1017/S003329172100146X. 33960286 PMC10324294

[6] M. J. Maiden , R. Trisno , M. E. Finnis , et al., “Long‐Term Outcomes of Patients Admitted to an Intensive Care Unit With Intentional Self‐Harm,” Anaesthesia and Intensive Care 49, no. 3 (2021): 173–182, 10.1177/0310057X20978987. 33853393

[7] M. Quesada‐Franco , M. D. Braquehais , S. Valero , et al., “A Comparison of Medically Serious Suicide Attempters Admitted to Intensive Care Units Versus Other Medically Serious Suicide Attempters,” BMC Psychiatry 22 (2022): 805, 10.1186/s12888-022-04427-8. 36536386 PMC9762004

[8] X. Walker , J. Lee , L. Koval , et al., “Predicting ICU Admissions From Attempted Suicide Presentations at an Emergency Department in Central Queensland,” Australasian Medical Journal 6, no. 11 (2013): 536–541, 10.4066/AMJ.2013.1730. 24348869 PMC3858606

[9] İ. G. E. İşcanlı , A. Özgültekin , and O. Ekinci , “Overview of ICU‐Accepted Suicide,” in *16th National Congress of the Turkish Society of Medical and Surgical Intensive Care Medicine & the 8th Euro‐Asian Critical Care Meeting*, November 13–16, 2019, Turkey, accessed September 24, 2025, https://search‐ebscohost‐com.lib.costello.pub.hb.se/login.aspx?direct=true&db=cin20&AN=141205627&site=ehost‐live.

[10] B. Şen and İ. Öztürk , “A Retrospective Evaluation of Suicidal and Accidental Drug Intoxication in Intensive Care Unit,” Cyprus Journal of Medical Sciences 7, no. 1 (2022): 48–52.

[11] D. T. Chung , D. Hadzi‐Pavlovic , M. Wang , S. Swaraj , M. Olfson , and M. Large , “Meta‐Analysis of Suicide Rates in the First Week and the First Month After Psychiatric Hospitalisation,” BMJ Open 9, no. 3 (2019): e023883, 10.1136/bmjopen-2018-023883. PMC6475206 30904843

[12] S. Fernando , D. Qureshi , M. Sood , et al., “Suicide and Self‐Harm in Adult Survivors of Critical Illness: Population Based Cohort Study,” BMJ 373 (2021): n973, 10.1136/bmj.n973. 33952509 PMC8097311

[13] M. Maple , L. M. Frey , K. McKay , S. Coker , and S. Grey , ““Nobody Hears a Silent Cry for Help”: Suicide Attempt Survivors' Experiences of Disclosing During and After a Crisis,” Archives of Suicide Research 24, no. 4 (2019): 498–516, 10.1080/13811118.2019.1658671. 31507236

[14] F. Shamsaei , S. Yaghmaei , and M. Haghighi , “Exploring the Lived Experiences of the Suicide Attempt Survivors: A Phenomenological Approach,” International Journal of Qualitative Studies on Health and Well‐Being 15, no. 1 (2020): 1745478, 10.1080/17482631.2020.1745478. 32223374 PMC7172699

[15] C. Dunkley , A. Borthwick , A. Bartlett , et al., “Hearing the Suicidal Patient's Emotional Pain,” Crisis 39, no. 4 (2018): 267–271, 10.1027/0227-5910/a00049. 29256270 PMC6137896

[16] J. C. Marshall , L. Bosco , N. K. Adhikari , et al., “What Is an Intensive Care Unit? A Report of the Task Force of the World Federation of Societies of Intensive and Critical Care Medicine,” Journal of Critical Care 37 (2017): 270–276. 27612678 10.1016/j.jcrc.2016.07.015

[17] M. Andersson , I. Fridh , and B. Lindahl , “Is It Possible to Feel at Home in a Patient Room in an Intensive Care Unit? Reflections on Environmental Aspects in Technology‐Dense Environments,” Nursing Inquiry 26, no. 4 (2019): e12301. 31273900 10.1111/nin.12301

[18] M. Meriläinen , H. Kyngäs , and T. Ala‐Kokko , “Patients' Interactions in an Intensive Care Unit and Their Memories of Intensive Care: A Mixed Method Study,” Intensive & Critical Care Nursing 29, no. 2 (2013): 78–87, 10.1016/j.iccn.2012.05.003. 23021148

[19] L. C. Stayt , K. Seers , and E. Tutton , “Patients' Experiences of Technology and Care in Adult Intensive Care,” Journal of Advanced Nursing 71, no. 9 (2015): 2051–2061, 10.1111/jan.12664. 25868064

[20] A. Tunlind , J. Granström , and Å. Engström , “Nursing Care in a High‐Technological Environment: Experiences of Critical Care Nurses,” Intensive & Critical Care Nursing 31, no. 2 (2015): 116–123, 10.1016/j.iccn.2014.07.005. 25442241

[21] N. E. Brummel and T. D. Girard , “Delirium in the Critically Ill Patient,” in Handbook of Clinical Neurology, vol. 167 (Elsevier, 2019), 357–375. 31753142 10.1016/B978-0-12-804766-8.00019-4

[22] Y. Lu , Y. W. Li , L. Wang , et al., “Promoting Sleep and Circadian Health May Prevent Postoperative Delirium: A Systematic Review and Meta‐Analysis of Randomized Clinical Trials,” Sleep Medicine Reviews 48 (2019): 101207. 31505369 10.1016/j.smrv.2019.08.001

[23] C. Madrid‐Navarro , R. Sanchez‐Galvez , A. Martinez‐Nicholas , et al., “Disruption of Circadian Rhythms and Delirium, Sleep Impairment and Sepsis in Critically Ill Patients. Potential Therapeutic Implications for Increased Light‐Dark Contrast and Melatonin Therapy in an ICU Environment,” Current Pharmaceutical Design 21, no. 24 (2015): 3453–3468. 26144941 10.2174/1381612821666150706105602

[24] I. Egerod , I. Bergbom , B. Lindahl , M. Henricson , A. Granberg‐Axell , and S. L. Storli , “The Patient Experience of Intensive Care: A Meta‐Synthesis of Nordic Studies,” International Journal of Nursing Studies 52, no. 8 (2015): 1354–1361. 25986960 10.1016/j.ijnurstu.2015.04.017

[25] L. Johansson , I. Bergbom , K. P. Waye , E. Ryherd , and B. Lindahl , “The Sound Environment in an ICU Patient Room — A Content Analysis of Sound Levels and Patient Experiences,” Intensive & Critical Care Nursing 28, no. 5 (2012): 269–279. 22537478 10.1016/j.iccn.2012.03.004

[26] L. Sellin , M. Asp , T. Kumlin , T. Wallsten , and L. Wiklund Gustin , “To Be Present, Share and Nurture: A Lifeworld Phenomenological Study of Relatives' Participation in the Suicidal Person's Recovery,” International Journal of Qualitative Studies on Health and Well‐Being 12, no. 1 (2017): 1287985, 10.1080/17482631.2017.1287985. 28245364 PMC5345596

[27] H. Arksey and L. O'Malley , “Scoping Studies: Towards a Methodological Framework,” International Journal of Social Research Methodology 8, no. 1 (2005): 19–32.

[28] M. D. J. Peters , C. Godfrey , P. McInerney , Z. Munn , A. C. Tricco , and H. Khalil , “Chapter 11: Scoping Reviews (2020 Version),” in JBI Manual for Evidence Synthesis, ed. E. Aromataris and Z. Munn (JBI, 2020), 407–452.

[29] M. J. Page , J. E. McKenzie , P. M. Bossuyt , et al., “The PRISMA 2020 Statement: An Updated Guideline for Reporting Systematic Reviews,” BMJ 372 (2021): n71, 10.1136/bmj.n71. 33782057 PMC8005924

[30] M. D. Peters , C. Marnie , A. C. Tricco , et al., “Updated Methodological Guidance for the Conduct of Scoping Reviews,” JBI Evidence Synthesis 18, no. 10 (2020): 2119–2126. 33038124 10.11124/JBIES-20-00167

[31] S. Bailey , “Critical Care Nurses' and Doctors' Attitudes to Parasuicide Patients,” Australian Journal of Advanced Nursing 11, no. 3 (1994): 11–17. 7980877

[32] Y. Kishi , H. Kurosawa , H. Morimura , K. Hatta , and S. Thurber , “Attitudes of Japanese Nursing Personnel Toward Patients Who Have Attempted Suicide,” General Hospital Psychiatry 33, no. 4 (2011): 393–397, 10.1016/j.genhosppsych.2011.02.005. 21762837

[33] J. Lönnqvist and J. Suokas‐Muje , “Staff's Attitudes Toward Patients Who Attempt Suicide,” Crisis 7, no. 1 (1986): 47–53. 3780272

[34] J. Suokas and J. Lönnqvist , “Work Stress Has Negative Effects on the Attitudes of Emergency Personnel Towards Patients Who Attempt Suicide,” Acta Psychiatrica Scandinavica 79, no. 5 (1989): 474–480, 10.1111/j.1600-0447.1989.tb10290.x. 2750548

[35] D. Wolk‐Wasserman , “The Intensive Care Unit and the Suicide Attempt Patient,” Acta Psychiatrica Scandinavica 71 (1985): 581–595, 10.1111/j.1600-0447.1985.tb02552.x. 4024974

[36] S. Moola , Z. Munn , C. Tufanaru , et al., “Chapter 7: Systematic Reviews of Etiology and Risk,” in JBI Manual for Evidence Synthesis, ed. E. Aromataris and Z. Munn (JBI, 2020).

[37] C. Lockwood , Z. Munn , and K. Porritt , “Qualitative Research Synthesis: Methodological Guidance for Systematic Reviewers Utilizing Meta‐Aggregation,” International Journal of Evidence‐Based Healthcare 13, no. 3 (2015): 179–187. 26262565 10.1097/XEB.0000000000000062

[38] J. L. Stollings , M. C. Balas , and G. Chanques , “Evolution of Sedation Management in the Intensive Care Unit (ICU),” Intensive Care Medicine 48 (2022): 1625–1628, 10.1007/s00134-022-06806-x. 35904562 PMC9334735

[39] M. D. Rudd , C. J. Bryan , D. A. Jobes , S. Feuerstein , and D. Conley , “A Standard Protocol for the Clinical Management of Suicidal Thoughts and Behavior: Implications for the Suicide Prevention Narrative,” Frontiers in Psychiatry 13 (2022): 929305. 35903634 10.3389/fpsyt.2022.929305 PMC9314639

[40] D. Darnell , A. Pierson , J. D. Whitney , et al., “Acute and Intensive Care Nurses' Perspectives on Suicide Prevention With Medically Hospitalized Patients: Exploring Barriers, Facilitators, Interests, and Training Opportunities,” Journal of Advanced Nursing 79, no. 9 (2023): 3351–3369, 10.1111/jan.15650. 36942775 PMC11334409

[41] P. Scott , P. Thomson , and A. Shepherd , “Families of Patients in ICU: A Scoping Review of Their Needs and Satisfaction With Care,” Nursing Open 6 (2019): 698–712, 10.1002/nop2.287. 31367391 PMC6650754

[42] L. Todres , K. T. Galvin , and K. Dahlberg , ““Caring for Insiderness”: Phenomenologically Informed Insights That Can Guide Practice,” International Journal of Qualitative Studies on Health and Well‐Being 9 (2014): 21421, 10.3402/qhw.v9.21421. 24461568 PMC3901386

[43] M. Vatne and D. Nåden , “Crucial Resources to Strengthen the Desire to Live: Experiences of Suicidal Patients,” Nursing Ethics 23, no. 3 (2016): 294–307, 10.1177/0969733014562990. 25539632

[44] K. P. Jackson , A. Welch , and S. Hopkinson , “Back From the Brink: The Experience of Hospital After a Suicide Attempt, and What Happens When You Go Home,” Issues in Mental Health Nursing 41, no. 7 (2020): 560–567, 10.1080/01612840.2019.1710010. 32357110

[45] S. Freud , “The Future Prospects of Psychoanalytic Therapy,” in The Standard Edition of the Complete Psychological Works of Sigmund Freud, vol. 11, ed. J. Strachey (Hogarth Press, 1957), 139–151.

[46] E. Betan , A. K. Heim , C. Zittel Conklin , and D. Westen , “Countertransference Phenomena and Personality Pathology in Clinical Practice: An Empirical Investigation,” American Journal of Psychiatry 162, no. 5 (2005): 890–898, 10.1176/appi.ajp.162.5.890. 15863790

[47] L. Michaud , K. T. Greenway , S. Corbeil , C. Bourquin , and S. Richard‐Devantoy , “Countertransference Towards Suicidal Patients: A Systematic Review,” Current Psychology 42 (2023): 416–430, 10.1007/s12144-021-01424-0.

[48] R. Weare , C. Green , M. Olasoji , and V. Plummer , “ICU Nurses Feel Unprepared to Care for Patients With Mental Illness: A Survey of Nurses' Attitudes, Knowledge, and Skills,” Intensive & Critical Care Nursing 53 (2019): 37–42, 10.1016/j.iccn.2019.03.001. 30878535

[49] C. A. Wilson , A. Gallo de Moraes , K. A. Cawcutt , V. Herasevich , O. Gajic , and K. Ramar , “Caring for Patients With Psychiatric Disorders in the ICU: The Role of Collaboration Between Critical Care and Mental Health Services,” Critical Care 26, no. 1 (2022): 1–4.34980198

[50] G. Rayner , J. Blackburn , K.‐l. Edward , J. Stephenson , and K. Ousey , “Emergency Department Nurse's Attitudes Towards Patients Who Self‐Harm: A Meta‐Analysis,” International Journal of Mental Health Nursing 28 (2019): 40–53, 10.1111/inm.12550. 30387232

[51] A. E. Helme , M. A. Bjørnaas , T. K. Grimholt , K. Hofsø , T. Rustøen , and J. Hagen , “Half Alive but Still Longing for Death: Exploring the Experiences of Patients Waking Up in the Intensive Care Unit After a Suicide Attempt – A Qualitative Study,” Australian Critical Care 39, no. 1 (2026): 101484, 10.1016/j.aucc.2025.101484. 41314156

